# Investigating skewness to understand gene expression heterogeneity in large patient cohorts

**DOI:** 10.1186/s12859-019-3252-0

**Published:** 2019-12-20

**Authors:** Benjamin V. Church, Henry T. Williams, Jessica C. Mar

**Affiliations:** 1 Department of Systems and Computational Biology, Albert Einstein College of Medicine, 1300 Morris Park Avenue, Bronx, 10461 NY USA; 2 Department of Mathematics, Columbia University, 2990 Broadway, New York, 10027 NY USA; 3 Department of Epidemiology and Population Health, Albert Einstein College of Medicine, 1300 Morris Park Avenue, Bronx, 10461 NY USA; 4 Australian Institute for Bioengineering and Nanotechnology, The University of Queensland, Brisbane, 4072 QLD Australia

**Keywords:** Skewness, Gene expression, Non-normality, TCGA, Cancer genomics

## Abstract

**Background:**

Skewness is an under-utilized statistical measure that captures the degree of asymmetry in the distribution of any dataset. This study applied a new metric based on skewness to identify regulators or genes that have outlier expression in large patient cohorts.

**Results:**

We investigated whether specific patterns of skewed expression were related to the enrichment of biological pathways or genomic properties like DNA methylation status. Our study used publicly available datasets that were generated using both RNA-sequencing and microarray technology platforms. For comparison, the datasets selected for this study also included different samples derived from control donors and cancer patients. When comparing the shift in expression skewness between cancer and control datasets, we observed an enrichment of pathways related to the immune function that reflects an increase towards positive skewness in the cancer relative to control datasets. A significant correlation was also detected between expression skewness and the top 500 genes corresponding to the most significant differential DNA methylation occurring in the promotor regions for four Cancer Genome Atlas cancer cohorts.

**Conclusions:**

Our results indicate that expression skewness can reveal new insights into transcription based on outlier and asymmetrical behaviour in large patient cohorts.

## Background

Typically, the analysis of gene expression data focuses on statistics that involve the mean and variance. The mean indicates the most representative value in the dataset, while the variance reflects how widely distributed the data points are. These two statistics are often used in combination for more sophisticated analysis, e.g. the t-test or linear regression. For most comparisons of transcriptomic datasets, functions of the mean and variance are sufficient for addressing a basic set of questions. Nevertheless, gene expression datasets are complex entities that represent an opportunity to move beyond simple hypotheses and, instead, ask questions that reflect deeper insights into the transcriptional regulation in cellular phenotypes.

The mean and variance are related to each other through the method of moments, where these two statistics represent the first and second estimators. Higher moments beyond the second one, have rarely been considered in the analysis of gene expression data, although some studies do exist [1–3]. This may be due in part to the requirement that datasets have large sample sizes since higher moments require more replicates to yield reliable estimates. In the case of gene expression studies, a scarcity of data has created a challenge for accurately estimating skewness. Limitations in technology can also affect the reliability of the data, e.g. due to batch effects, the handling throughput of samples, and lack of standardization to correct for noise or technical artifacts.

Why might skewness be considered an informative parameter to study for understanding gene expression? When identifying transcriptional regulatory programs, understanding trends in data that span the tail ends of the distribution have helped to identify important regulators. For example, expression quantitative trait loci (eQTL) mapping has contributed to understanding genetic variation and regulation [4, 5]. Reliable detection of eQTLs is based on a linear model and therefore predicated on identifying genes that span large ranges in gene expression for individuals from different allele groups of a gene [6–8]. Because skewness is a statistic that directly models outliers, gene expression skewness, therefore, represents a valuable way to understand the distribution structure of a population of cells or patients. More specifically, skewness is a property that reflects the asymmetry in size and length of the two tails of a distribution. Given that the tails of a probability density distribution reflect the most extreme data points, it seems plausible that measures based on skewness would be useful for identifying transcriptional regulators. For studies related to precision medicine, skewness represents a potential avenue towards identifying the genes that show the greatest variation in the population.

As we begin to recognize the importance of non-Normal distributions in genomics [9], it follows that skewness is also emerging as a significant measure of interest. For example, in RNA-sequencing datasets, transcript read counts are assumed to follow a Negative Binomial or Poisson distribution [10, 11]. In single cell datasets a range of different distributions are used for modelling gene expression; e.g., multi-modal, asymmetric, and highly skewed distributions [12, 13]. In short, skewed distributions are becoming more recognized for their utility for modelling transcriptomes.

A limited number of studies have attempted to investigate the utility of skewness in biology. In 2012, Casellas and Verona [1] modeled the prevalence of skewness in human transcriptomes. However, the study was limited by several features of its design. Specifically, the sample sizes of the datasets used were too small to obtain a stable estimate of skewness. Additionally, different microarray platforms were used for each of the four datasets included in their study, making it difficult to differentiate the influence of technology-specific effects from genuine patterns of expression skewness. The study also failed to address how expression skewness differed for data collected from RNA-sequencing. A very early study also investigated skewness in single cells where gene expression profiles were modeled using a lognormal distribution. However, the focus of this study was limited to identifying departures from normality of the transformed data [13]. Another study also assessed the validity of the Normal distribution for cancer gene expression datasets, demonstrating how heavy-tailed distributions that included high degrees of skewness and kurtosis were appropriate alternatives for modeling these datasets [2].

**Fig. 1:**
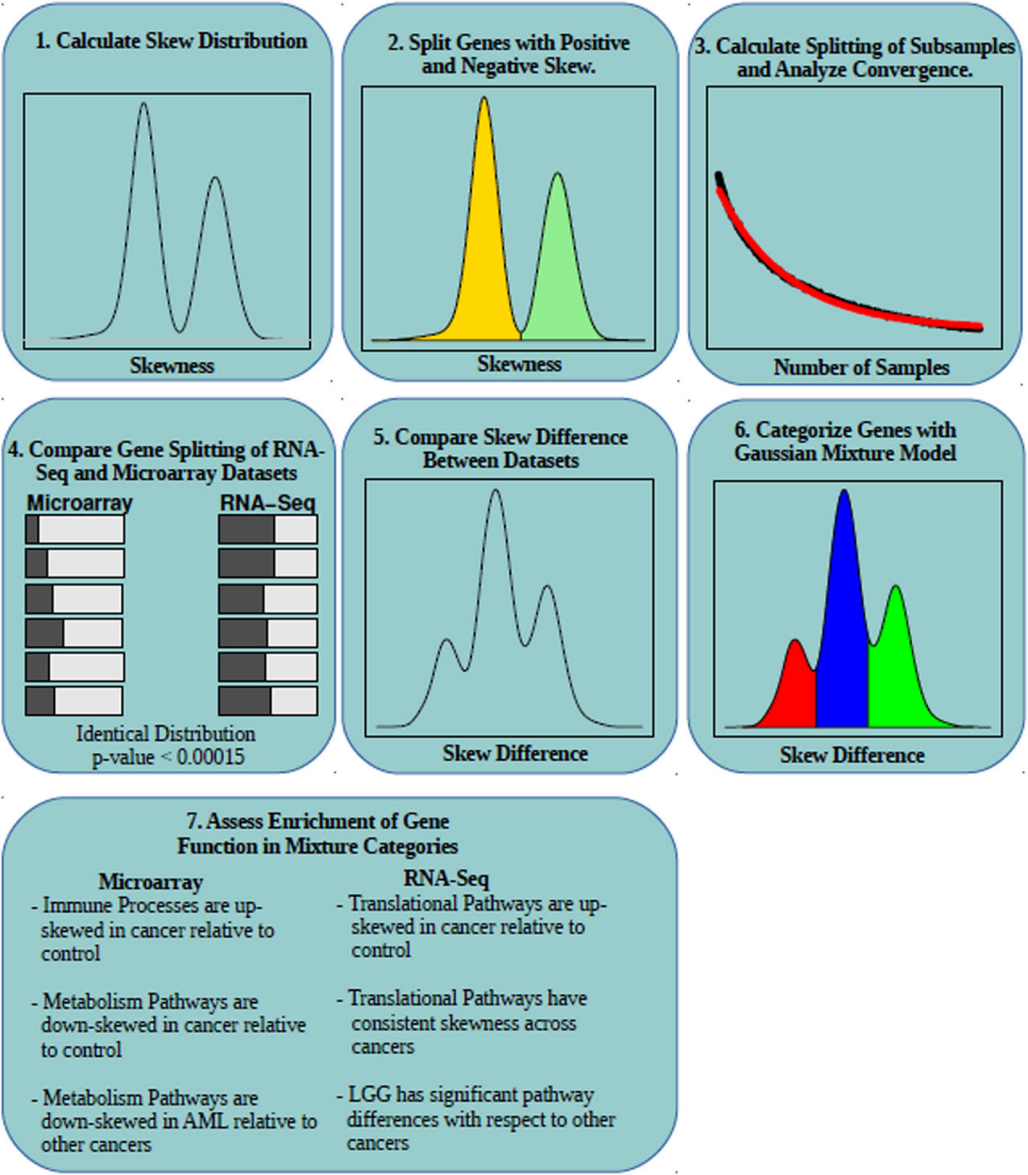
Outline of gene expression skewness analysis. This cartoon depicts the main steps involved in the analysis of gene expression skewness conducted in this study

Our study breaks new ground by providing a thorough investigation of skewness for a comprehensive set of gene expression datasets that have been generated by both microarrays and RNA-sequencing technologies (Fig. 1). The results of this study identified an increase in positive skewness for immune-related pathways in cancer versus control datasets. We also examined the relationship between expression skewness and differential DNA methylation for four Cancer Genome Atlas (TCGA) patient cohorts and identified genes that were significantly correlated. The robustness of this relationship was most evident for loci in the promotor regions. Collectively, the results of this study indicate that regulatory insights can be extracted by investigating gene expression skewness for large patient cohorts.

## Results

***Measuring skewness of gene expression.***

The skewness metric used in this study was calculated by dividing the cube root of the third moment of a distribution by its standard deviation. Explicitly, the relative skewness of a gene’s transcription expression (*g*) over a population of size |*X*| is defined as

1 $$ S_{g}(X) = \frac{1}{\sigma_{g}} \sqrt[3]{\frac{1}{|X| - 1} \sum_{x \in X} (g_{x} - \mu_{g})^{3}}  $$

This statistic was selected because of its ability to differentiate between distributions containing wide, slightly asymmetric shapes and those that were narrow and highly asymmetric. Although this statistic is a biased sample estimator of the population skewness, an unbiased statistic can be constructed by multiplying the third moment by the correction factor |*X*|/(|*X*|−2) [14].

Asymptotically, as the population size increases, this correction tends to 1 and bias of Sg(X) disappears. For a dataset with X = 500 samples, this correction is of the order of 0.2%, resulting in a negligible change to the skewness calculation. It is worth highlighting that one of the disadvantages of using the third moment on its own as an estimate of skewness, is that it cannot readily distinguish between two qualitatively different distributions (Additional file 1: Figure S1). Hence, for our study, the metric *S*_*g*_(*X*) was used to investigate skewness of gene expression.

***Application of expression skewness to a diverse range of studies representing six microarray and six RNA-seq datasets.***Both microarray and RNA-sequencing datasets were included in this study to avoid any biases that may be apparent with one technology but not the other. A total of six microarray datasets were used, and these datasets represent five cancer patient cohorts and one control cohort. An ovarian serous cystadenocarcinoma (OV) group (568 patients), a glioblastoma multiforme (GBM) cohort (548 patients), and a Luminal A (LumA) breast cancer subgroup (284 patients) were taken from TCGA. The TCGA-based datasets were profiled using Agilent 244K Custom Gene Expression Microarray (G4502A-07-3).

An additional three microarray datasets were taken from the NCBI Gene Expression Omnibus (GEO). We used two cohorts with acute myeloid leukemia (AML), one exclusively with individuals over the age of 60 (GSE6891) (461 patients) with samples collected from both blood and bone marrow and the second (GSE15434) of exclusively normal karyotype (NK) AML (251 patients) with samples collected from mononuclear cells. These two datasets were profiled using an Affymetrix Human Genome U133 Plus 2.0 Array. The control for the microarray group was a HapMap expression profiling (GSE6536) collected via a Sentrix Human-6 Expression BeadChip.

A total of six RNA-seq datasets were included in this study, five cancer cohorts and one control cohort. The cancer cohorts included skin cutaneous melanoma (SKCM) (470 patients), head and neck squamous cell carcinoma (HNSC) (519 patients), lower grade glioma (LGG) (514 patients), lung squamous cell carcinoma (LUSC) (495 patients), and kidney renal clear cell carcinoma (KIRC) (531 patients). The RNA-seq control dataset was sourced from 465 lymphoblastoid cell lines from the 1000 Genomes project created by the Geuvadis consortium [15] (ArrayExpress accession id E-GEUV-1).

***Microarray and RNA-seq datasets have different proportions of genes with positively-skewed and negatively-skewed distributions.***

**Fig. 2:**
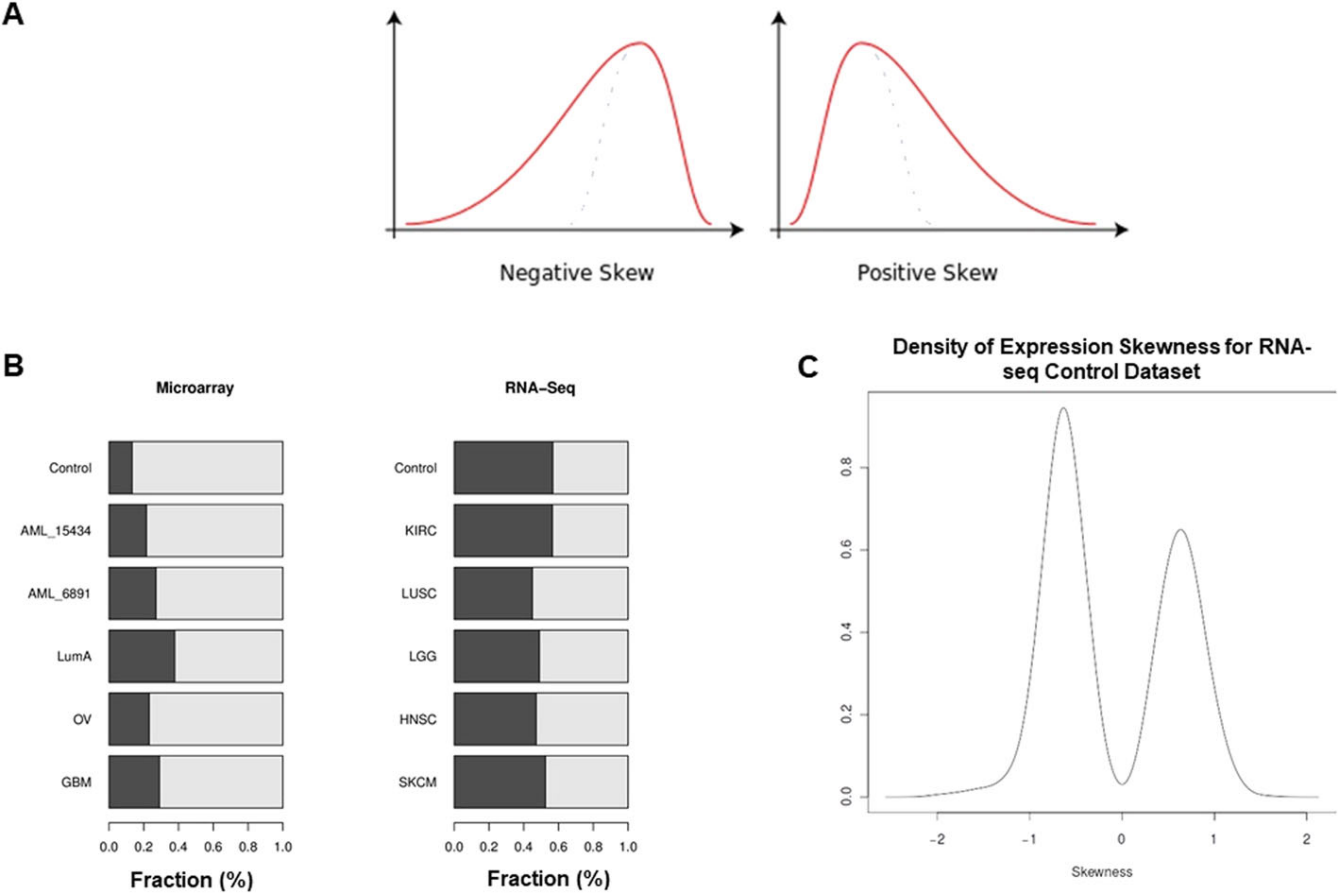
Skewness in the gene expression study. **a**. Contrasting negative and positive skewed distributions. **b**. The proportions of genes with negative and positive skewed expression reflect differences between microarray and RNA-Seq datasets. The dark grey bars indicate the fraction of genes with a negatively-skewed expression distribution and the light grey bars indicate the remaining fraction of genes with positively-skewed expression distribution. **c**. Distribution of gene expression skewness for the RNA-seq control dataset

The comparison of both microarray and RNA-seq datasets allowed for an investigation into how skewness was distributed in data from two different gene expression technology platforms. The comparison between the two selected platforms allowed for the opportunity to identify platform-specific changes in skewness across multiple cancer-related datasets. The skewness metric reflects the degree of asymmetry in a gene’s expression profile within a patient cohort (Fig. 2a). Genes were divided into two mutually exclusive groups based on whether their skewness expression profile reflected positive or negative skew. We defined gene splitting as the percentage of genes that were allocated to these positive and negative skew groups. The number of genes with negatively-skewed versus positively-skewed gene expression distributions varied between the twelve gene expression datasets (Fig. 2b). On average, the degree of gene splitting differed substantially between the microarray and RNA-seq datasets (i.e. 0.253 in microarray datasets versus 0.511 in RNA-Seq datasets). The results indicate that very few genes had zero skew in any dataset, i.e. few genes have symmetric gene expression distributions (Fig. 2c).

We tested the two sets of gene splits between the RNA-seq and microarray datasets using Welch’s t-test and found that there is adequate evidence to reject the null hypothesis that the two sets are from the same distribution (P-value <0.00015). The Shapiro-Wilkes tests supported the normality assumption for both microarray (P-value <0.976) and RNA-Seq gene splits (P-value <0.480) which was necessary to apply the t-test. A possible explanation for why the microarray and RNA-seq platforms generated gene splits that were different may lie with the fact that microarrays are less able to detect low transcript reads. Therefore, the left tails of expression distributions are more likely to be attenuated in microarray data and result in fewer negatively skewed genes. This effect aligns with the observation that there is an under-representation of negatively skewed genes in the microarray datasets (Fig. 2b).

For TCGA datasets, known batch effects were adjusted for in the microarray and RNA-seq datasets to prevent any biases due to known batches. The method of batch correction used for TCGA microarray data was based on median and standard deviation correction, following Hsu et al. [16], and for TCGA RNA-seq data, a linear model using limma [17]. Batch correction for the remaining GEO datasets was not possible due to information on potential batches being unavailable. However, with the batch correction applied to both types of TCGA gene expression data, it is unlikely that batches effects are contributing to the differences in skewness gene splits observed between microarray and RNA-seq datasets.

***Sample size calculation demonstrate robustness of the gene expression skewness results.***

Reliable inferences come from ensuring that an adequate sample size is used in the analysis. This is just as critical for measuring skewness. We analysed the effect of sample size on the fraction of genes with negative skewness as a surrogate for the sensitivity of individual skewness results to sample size (Additional file 2: Figure S2). The gene splitting statistic was computed on random subsamples of the various datasets. The gene splitting is organized into a sequence indexed by increasing sample size. Two metrics were used to assess the convergence of each sequence, the rate of convergence and the error to the limit. For an infinite sequence *s*_*n*_ with limit *L*, the rate of convergence (C) is a number between zero and one defined as

2 $$ C = {\lim}_{n \to \infty} \frac{L - s_{n+1}}{L - s_{n}}  $$

While the *n*^th^ error (E) is defined as

3 $$ E_{n} = \frac{s_{n} - L}{L}  $$

Because only a finite section of the sampling sequence is known, the limits cannot be computed directly. Therefore, we assumed that the convergence can be approximated by an exponential decrease of the form: *s*_*n*_=*ae*−−*bn*+*L* with constants *a*, *b*, and *L*. The constants were estimated using least-squares regression from the data. Once these constants were determined, the limit of the sequence is approximately L and the rate of convergence is given by *e*−*b*.

The errors in the gene splitting limits are well within acceptable ranges (0.025 to 1.136, based on absolute percent error, see Additional file 4: Table S1, Additional file 2: Figure S2). The rates of convergence that were close to 1 suggest that either the convergence is very slow for these cases, which further supports our choice to include only large datasets, or that the assumption of exponential convergence is not applicable and instead sub-linear convergence (such as a power law) is more appropriate. Nevertheless, the size of the error margins allows us to accept results based on these datasets.

***Contrasting gene expression skewness between two datasets identified gene sets corresponding to three different types of skewed relationships.***

To investigate how changes in asymmetry of gene expression may identify new insights into phenotype, we compared how skewness changed between two datasets by examining the distribution of the difference in gene expression skewness (Additional file 3: Figure S3A). For each gene, its skew difference is simply the signed difference of its skewness in each of the two datasets being compared. We adopted the convention that a positive skew difference occurs when a gene has a greater skewness in the first mentioned of the two datasets being compared. The skew difference distributions are qualitatively multi-modal and thus lend themselves to discrete categorization (Additional file 3: Figure S3B). We used a variable standard deviation Gaussian mixture model [18] to cluster genes by skew difference. K = 3 mixture components were chosen to maximize the Bayesian Information Criterion (BIC) over the range 1 to 10 components and to fit the qualitative observation of three independent modes (Additional file 3: Figure S3C).

***Pathway over-representation analysis identified immune-related pathways had increased skewness in cancer microarray datasets relative to control, whereas metabolic pathways had decreased skewness.***

**Fig. 3:**
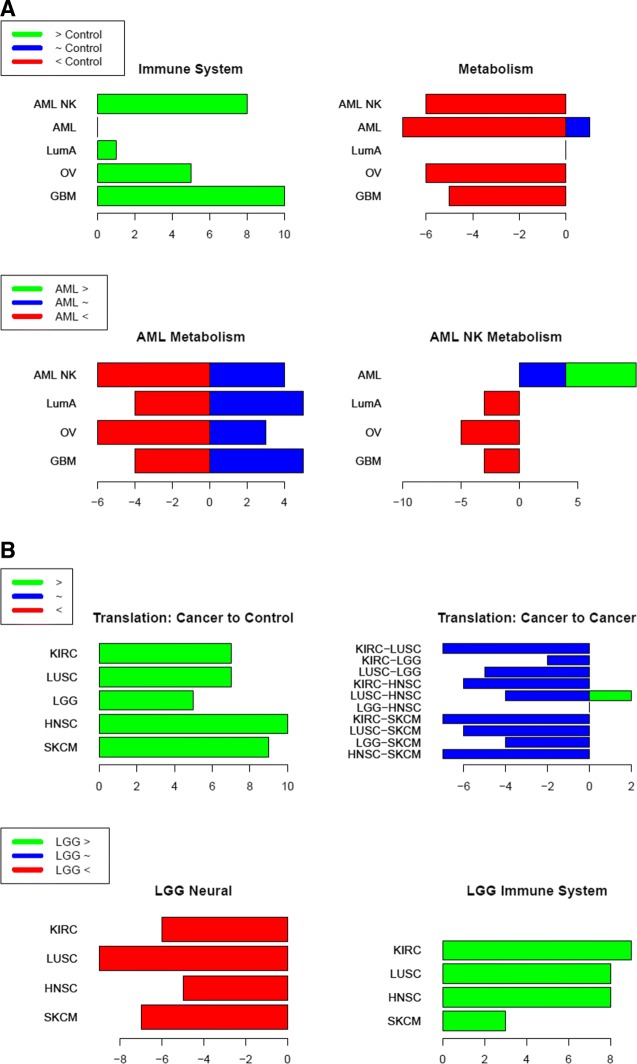
Pathway trends for skewness comparisons between datasets. **a**. Results are shown for microarray dataset comparisons. **b**. Results are shown for RNA-seq dataset comparisons. The bars represent the number of category-specific pathways that appear in the ten most significant pathways of the colour-specified group. “ >” refers to genes where there was an increase towards more positive skew, “ ∼” refers to genes that had negligible change in skew between the datasets, and “ <” refers to genes where there was an increase towards more negative skew. For **a**. in the Immune System and Metabolism plots, green refers to genes that have a greater skew in each cancer on the y-axis as compared to the control. In the AML Metabolism plots, green refers to genes that have a higher skew in AML compared to each cancer on the y-axis. Large red scores in Metabolism suggest that metabolic pathways have a lower skew in cancers compared to control. However, red scores in AML Metabolism suggest that metabolic pathways in AML have a lower skew than those in other cancers. For **b**. In the Translation plots, green refers to genes that have a greater skew in each cancer on the y-axis as compared to the control/other cancer on y-axis. In the LGG plots, green refers to genes that have a higher skew in LGG compared to each cancer on the y-axis. **b** Overview of DNA methylation and gene expression skewness analysis. This cartoon outlines the main steps for investigating the relationship between expression skewness and DNA methylation in four TCGA datasets

Immune pathways consisted of up-skewed genes from microarray cancer datasets compared with control. Metabolic pathways had a lower or more negative skew in cancer, with respect to control (Fig. 3a). However, LumA is an outlier in the metabolic category showing a small but non-negligible effect in the opposite direction. Immune pathways had almost no enrichment in any skewness group when comparing cancers. This suggests that if immune system pathways are truly more highly skewed across cancer patients, this skew is not consistent between different cancers. Nor is there a solid relationship between the cancer type and the skew. However, the cancer to cancer comparison does provide insight into the skew of the metabolic pathways. Both AML groups showed lower skews than other cancers in metabolic pathways. Furthermore, normal karyotype AML has a higher skew in metabolic pathways than standard AML.

***Tissue-specific comparisons of RNA-Seq datasets also point to an increased skewness in other cancers versus LGG for immune-related pathways.***

In addition to the cancer versus normal comparison, the nature of the RNA-sequencing datasets allowed for comparisons between cancers to identify tissue-specific effects in gene expression skewness (Fig. 3b). Most strikingly, LGG has consistently greater skew for immune system pathways and lower skew for pathways related to neural tissue than the other cancers used in this study. It was also noted that pathways related to translation, including protein synthesis and targeting to the endoplasmic reticulum, are consistently more positively skewed in the cancer groups as compared to control. Translation has the highest total score (39) across cancer to control comparison of any group considered. Furthermore, comparisons between cancers show that translational pathways are almost entirely comprised of genes whose skewness is consistent between cancers. These results suggest that the skewness of translational genes in tumour transcriptomes is consistent between cancers and greater than that of healthy tissue.

***Investigating the relationship between skewness and mean gene expression suggests that these two statistics are generally independent.***

The skew measure developed in this study was designed to be shift-invariant and scale-invariant. The measure therefore does not depend on the mean or standard deviation. In principle, this means that there should not be a bias or relationship between the skewness measure and the expression of the gene, including the mean. Because the skewness measure used in this study was designed to be independent of differences due to shift and scale of a gene’s expression distribution, we were able to isolate the effects due to skewness as estimated by this measure directly. Plots showed the relationship between skewness and mean gene expression for genes in the RNA-seq datasets (Additional file 1: Figure S1) and microarray datasets (Additional file 2: Figure S2). Overall, these plots indicate that for much of the mean expression spectrum, there is minimal correlation with the skewness measure. Some correlation exists for very lowly-expressed genes (Additional file 1: Figure S1A, log2(expression) <1). The relationship between genes that were differentially expressed and skewed differently between datasets is an area of investigation that may further identify the regulatory information captured by the skewness metric.

***Correlation between differential DNA methylation and expression skewness involved first identifying the patients lying in the tails of the gene expression distribution.***

Four DNA methylation datasets from TCGA patient cohorts whose expression data were previously analysed in this study were used to investigate the effect of epigenetic regulation on gene expression skewness (Fig. 3). These data correspond to patients from the SCKM, LGG, LUSC, and KIRC patient cohorts. Methylation data for other groups notably HNSC were unavailable. Our analysis hinges on comparisons between patients falling in the tail of a specific gene expression distribution versus those patients in the remainder of the distribution. Because the tail of a distribution is not a precisely defined concept, we examined two alternative methods of determining the tail and non-tail regions of a distribution and demonstrate that, with an appropriate choice of parameters, these methods produce similar results (Additional file 3: Figure S5).

First, we examined a method (referred to as quantile splitting) that takes the most extreme samples in the direction to which the distribution is skewed and employs a specific quantile cut-off to ensure that tail area is constant across all distributions ensuring that our statistical tests are run over equal sized samples. Second, we considered a Gaussian splitting method in which the central mode is fit by a Gaussian density function using the R package mclust [18]. Points that deviate significantly from the fit density in the direction of the distribution skew make up the tail. Simulations demonstrated that the features of tails identified by these two methods are qualitatively very similar (Additional file 3: Figure S6). When the two methods were run on real data, the two methods produced an overlap of 88% significant genes. Based on these tests, we opted to use the quantile splitting approach since it had almost identical results with the Gaussian splitting method but avoided some pathological situations that were potentially possible.

***Comparing the most extreme differential DNA methylation patterns in the tail of the gene expression distributions highlighted for changes in gene expression skewness for four TCGA datasets.***

With identified tail and non-tail regions of each gene expression distribution, we used a Wilcoxon signed rank test on the M-values for each probe annotated to that specific gene of patients who fall into the tail and non-tail groups to test the hypothesis that the DNA methylation status of all patients are drawn from the same population. After correction for multiple testing using the Benjamini-Hochberg procedure [19], genes were ranked by the P-value of their most significant probe and the percentage of methylation probes annotated to them which were significant (P-value <0.01).

**Fig. 4:**
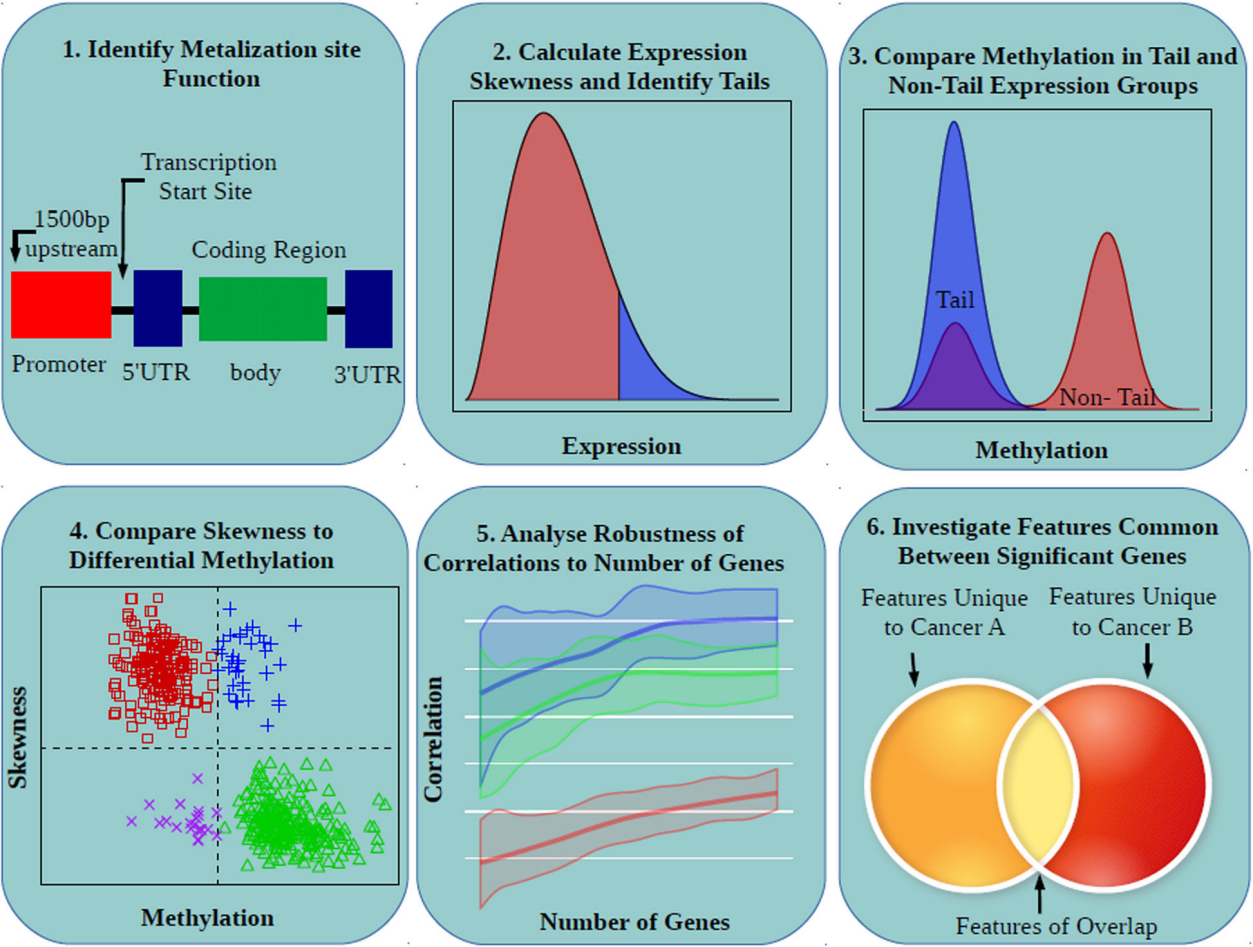
Overview of DNA methylation and gene expression skewness analysis. This cartoon outlines the main steps for investigating the relationship between expression skewness and DNA methylation in four TCGA datasets

In order to look for relationships between differential methylation in the tail and non-tail expression regions and the skewness of the expression distribution, we generated plots showing the 500 most significant genes (with respect to their differential DNA methylation) plotted by their skewness vs difference in M-value between the tail and non-trail regions (Fig. 4). Plots are distinguished based on the functional region of included probes. We separated the data into quadrants based on positive/negative skew and increase/decrease in methylation. These quadrants follow the qualitative clustering of the data. We used Fisher’s exact test to test differences in number of data points that fall within each quadrant between the categories of probe functional regions.

For each of the four TCGA datasets, we identified genes that had a statistically significant correlation between expression skew and differential DNA methylation (P-value <0.01). For the KIRC cohort, there were 2023 significant genes, 7782 genes in the LUSC cohort (Additional file 3: Figure S7), 8500 genes in the SKCM cohort (Additional file 3: Figure S8), and 9053 genes in the LGG cohort (Additional file 3: Figure S9). It is important to highlight that this correlation between gene expression skewness and DNA methylation focused only on the genes that mapped to the top 500 most significant differentially methylated and cannot be extrapolated to general correlations across the genome or methylome.

***Assessing the robustness of the correlation between differential DNA methylation and gene expression skewness identified the relationship for the promoter regions as being the most robust.***

**Fig. 5:**
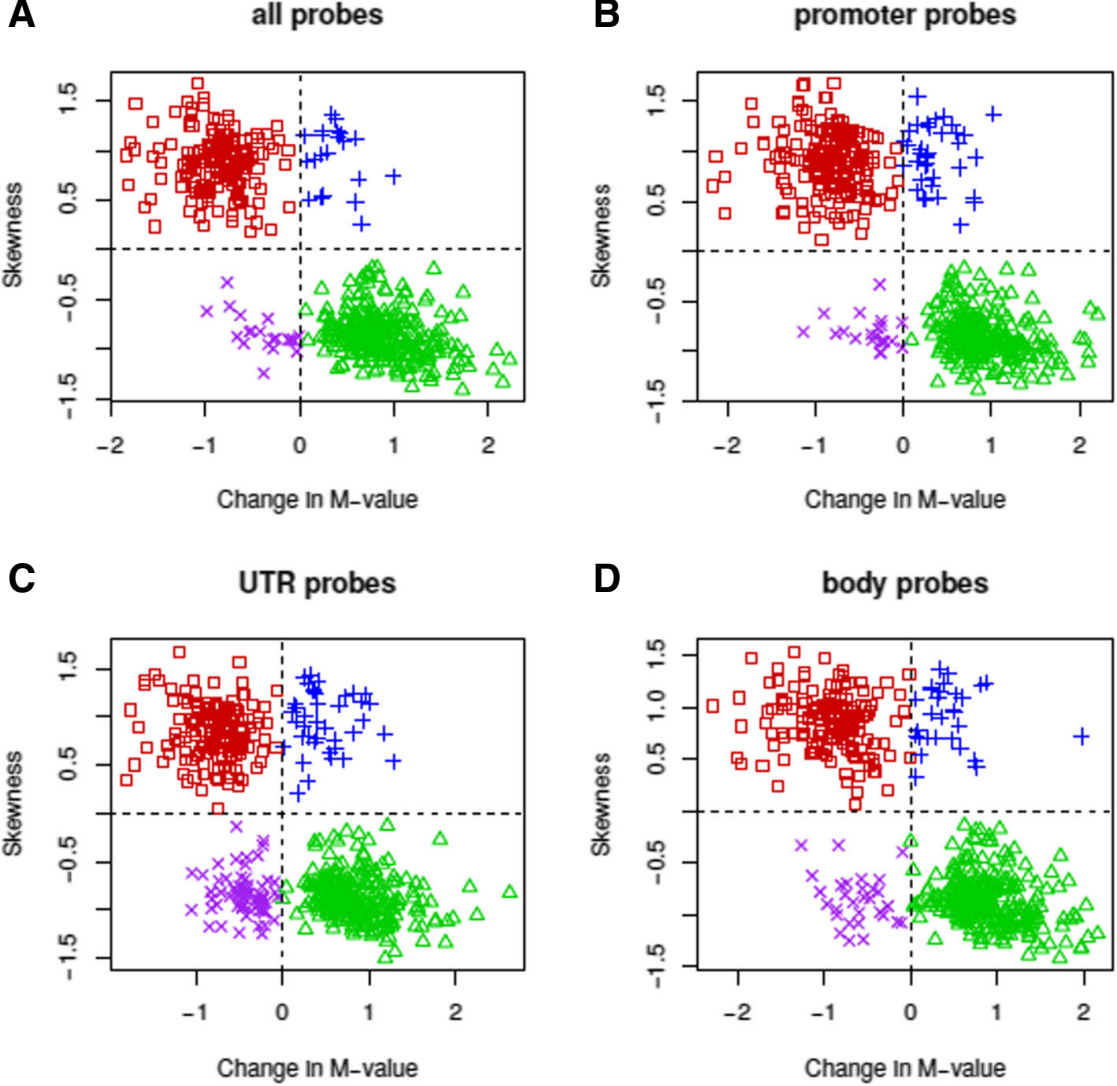
The relationship between expression skewness and differential Methylation in KIRC. The gene expression skewness for the 500 most significant genes identified from the KIRC cohort are plotted against the average change in DNA methylation of their significant annotated methylation sites classified by functional region for **a**. all probes, **b**. promoter, **c**. UTR, **d**. gene body. The data are split into quadrants representing positive/negative skewness and increase/decrease in methylation. Red points = upper left quadrant, blue = upper right, purple = lower left, green = bottom right

We also tested the robustness of these results when the number of significant genes included in the correlation test was altered (Fig. 5). The Pearson correlation coefficient between differential methylation and skewness and its associated 95% confidence interval is calculated for each plot. We assessed the dependence of our correlation results on the arbitrary choice of selecting the 500 most significant genes by graphing the correlation coefficients for probes based on function as we increased the number of significant genes included to highlight the most robust features of the correlation results. Plots show the fitted smoothing spline of the data (using the R smooth.spline function).

When fewer significant genes were included, it was clear that for all probe locations (gene body, promoter, UTR), the degree of correlation moved towards more extreme negative values. However, for the promoter regions in both KIRC (Fig. 5a) and SKCM (Fig. 5b), the correlation profile remains almost invariant for the range of significant genes sampled (100 to 500 genes). The most significant and highly negatively correlated methylation sites in non-promoter regions are washed out by genes that fall on the increasing diagonal (Fig. 5, see blue and purple regions). The rapid loss of correlation in non-promoter regions as more genes are included is especially apparent in the SKCM cohort (Fig. 5b).

***Genes with significant correlation between expression skewness and differential DNA methylation were enriched for cancer-related and immune-related pathways.***

Using MSigDB and Gene Ontology (GO) terms, we investigated whether the sets of genes with a significant correlation between expression skewness and differential DNA methylation datasets were enriched for specific pathways. We took the intersection of the most significant genes across the 4 methylation datasets and the top 1269 significant genes from each dataset that did not fall into the intersection. 1269 was chosen, as the KIRC dataset had the fewest unique significant genes and we wanted equal numbers of genes to test for side-by-side comparison. These sets of genes were used to query the C4 (computational genes) and C6 (oncogenic signatures) datasets from MSigDB [20]. Additionally, these sets of genes were also used to query several GO pathway analysis tools (Gorilla [21], PantherDB [22]). Due to the challenges of finding significant patterns from the results of these queries, we adopted three methods of analysis: looking for variation from the baseline set of genes, looking for variations between cancers, and investigating uniquely significant genes defined as significant genes that appear in one cancer cohort but do not appear in the overlap.

These trends seem to be comprised of two major trends; first, enrichment in immune response, shown by the leukocyte activation GO terms and the significant overlap with the gene set MODULE_84, comprised of genes related to immune response (Additional file 3: Figure S9). Second, enrichment in cancer hallmarks [23], shown by the GO terms related to angiogenesis and signal transduction [24] as well as significant overlap with the cancer genes in MODULE_55 (see Additional file 5: Tables S2, Additional file 6: Table S3).

## Discussion

When looking at samples of data extracted for a population, the goal of statistics is to learn about the distribution of samples by approximation. Mathematically, we can only ever approach a distribution through models, and each moment of a distribution adds a new dimension to that model. The first moment, the mean, tells us about the middle of a distribution. The second moment, the variance, tells us about the normal part of a distribution. The third moment, skewness, tells us about the outliers of a distribution. These outliers have the potential to teach researchers quite a bit about cancer, especially in regard to cancer hallmarks, and also in learning why patients in a study have exceptionally positive or negative outcomes. In this way, skewness can provide a new angle that is distinct from insights learned from differential expression. For example, the gene ARHGAP30, which codes for Rho GTPase-activating protein 30, is a pivotal regulator for p53 acetylation and functional activation in colorectal cancer [13, 25]. Our analysis shows that ARHGAP30 exhibits notable differential skew between cancer and control cohorts (Additional file 3: Figure S10).

Variation in genetics and cancer biology has been the driving component in discoveries of how the genome and transcriptome function. Our results suggest that skewness has the potential to bring us closer to more authentic comparisons about the tails of distributions that cannot be covered by lower statistical moments, which are typically the status quo tools for analysis in computational biology. Significantly, we have seen enrichment in genes and GO terms that are sensible reflections of the specific datasets from which they come (e.g., neuro tumour clinical annotations in LGG, Additional file 3: Figure S10). This demonstrates that our method uncovers real biological effects and epigenetic features from matched DNA methylation analysis.

A recurring theme in our research has been that both immune and metabolic processes make up a large portion of the top set of significant results, as it relates to features of genes identified through either their significance due to skewness or due to epigenetic regulation. We explored this recurrence in two directions: first, there is a growing body of research into the importance of reprogramming cellular metabolism to the genesis and proliferation of tumour cells [26–28]. Furthermore, cellular metabolic pathways signal regulatory enzymes and nearby cells which aids in the oncogenic reprogramming of metabolism and shift in expression profiles of metabolic related genes [29]. There is also precedent for differential expression of immune related genes in tumours, an effect caused by the cancer immune response. This effect most notably explains the prevalence of terms such as lymphocyte activation in our results [30].

However, mixtures of cell types present in the samples used to produce expression data may lead to immune or metabolic genes being enriched in results. This occurs not because of patterns in the data, but because of the nature of skewness analysis. We predict that this effect would be strongest in pathways related to the immune system because the specialization of immune cells and differences between patients of present cell types may increase the size and directionality of expression tails for immune-related genes. These tails reflect variability among cell types rather than among patients. This effect should be investigated formally by studying datasets in which the proportions of different immune cells have been captured and evaluating whether differences in skewness track with those different proportions.

An especially informative study might look at a population of cells with homogeneous cellular function, perhaps immune specific cells such as leukocytes, whose sequencing data is available from the social genetics of loneliness study from UCLA [31]. We hypothesise that immune system-related pathways would show lower significance to skewness analysis in a more homogeneous cell population if cell specialization is, in fact, the driver of our results. This raises the utility of applying skewness to single cell populations as an informative way to understanding transcriptional regulation. Limitations of lower moments have been observed in single cell gene expression modelling where average expression is carrying only limited information. There is movement towards modelling either changes in distribution shape or simply recognizing that genes do not have similar distributions across the cell population [12]. We present skewness analysis as a candidate method for researchers aiming to investigate the gene expression distribution shape in single cell applications.

Our results regarding the distribution of skewness across the genome highlight a striking difference between microarray and RNA-seq analysis that is non-obvious to other statistics. The much lower sensitivity of microarray technologies to low-transcript signals manifests itself as an attenuation of leftward tails in gene expression distributions. This attenuation vastly reduces the number of genes that are identified by microarray analysis as having negative skewness across their gene expression population. We determined that this inadequate spectrum makes skewness analysis applied to data collected via microarrays unreliable due to the necessity of sensitivity at broad range of expression values in both the high and low transcript regimes.

We further concluded that large sample size is critical to draw accurate and robust conclusions from skewness analysis. The clear trend of uniform divergence of skewness results from the limiting case as fewer samples are included in the calculations highlights. Care must be taken to ensure results dependent on higher moment calculations are not sample size-dependent, as can be tested by random down-sampling. Based on our own down-sampling calculations, we recommend that skewness results be applied to datasets of at least 200 samples to ensure the robustness of any conclusions. Future experimental design of skewness analysis must consider both the need for large datasets and also the necessity of a measuring platform that allows for capture of a broad spectrum of expression reliably.

Our analysis of the link between DNA methylation and skewness has led to interesting and somewhat unexpected results. These results have implications both for the biological interpretation of skewness and for the study of mechanisms behind the regulatory effects of DNA methylation. Primarily, our conclusion suggests promoter methylation, known to be an epigenetic downregulation of gene expression, is a driver of skewness. Skewness is negatively correlated with tails exhibiting increased methylation of promoter regions. The fact that the promoter probes have the most extreme correlation between methylation and skewness, persisting even with sampling of larger significant genes, suggests that there is a mechanistic or regulatory event that skewness is identifying by integrating these two data types. This correlation was strong (0.8) across all cancer cohorts studied and robust to the number of included genes. The consistency in the negative direction of correlation for all three functional groups of methylation probes also supports the idea that skewness is linked to, or is impacted by, epigenetic signatures.

Furthermore, the number of genes identified as having significant differential methylation between their tail and non-tail regions (at least 2000 in every cohort at the P-value <0.01 level) further supports the idea that skewness is epigenetically driven. We hypothesize that deviations between the methylation of loci between patient sub-groups creates tail regions. The relative sizes of these sub-groups determine the asymmetry and therefore the skewness of the gene expression distribution. It remains unknown whether skewness has genetic drivers as well as epigenetic ones. We propose further study into the difference in genetic mutations, specifically single nucleotide polymorphisms, exhibited by patients in the tail for gene expression.

Surprisingly, we found that highly significant methylation probes, regardless of regulatory function, showed a strong negative correlation between differential methylation and skewness. This suggests a more direct effect between changes in gene expression being influenced by DNA methylation at functional locations in or near genes. The current understanding of methylation holds that methylation loci in the gene body have an upregulatory effect with increased methylation counter to these results. This may be indicative of unknown mechanisms that allow DNA methylation in non-promoter regions to downregulate gene expression. We suggest that skewness analysis offer a new method for studying methylation that highlights certain highly specific effects, such as the overall negative correlation between differential methylation and distribution asymmetry. However, while these results are interesting, the promoter results are in line with how we understand the effects of DNA methylation at that region of the genome and their corresponding effect on gene expression.

The observation that negative correlations were exhibited between DNA methylation in the gene body and UTRs and the expression of significant skewed genes was more surprising. However, negative correlations have been reported in some instances. This may suggest a subset of gene-loci have a specialized or less generic function. And the consistency of this specific trend across the four different cancer types that were investigated suggests that it may be a shared feature of cancer more generally. The proposed specialization of these gene-loci provides justification for our study of common features between these genes.

The influence of tumor purity on the gene expression skewness distribution may be interesting to investigate further, especially in relation to the pathway over-representation analysis. In a previous study, we have addressed how tumor purity is associated with the shape of gene expression distribution using TCGA datasets [38]. de Torrente et al. investigated the extent to which tumor purity was correlated with gene expression for GBM and OVC TCGA cohorts from both microarray and RNA-seq datasets. For GBM (microarray), OVC (microarray), and OVC (RNA-seq), a very small number of genes had significant correlation with tumor purity (377 to 441 genes, adjusted P-value <0.01) and for GBM (RNA-seq), there were zero significant correlations. Both GBM and OVC microarray datasets were included in the skewness study. De Torrente et al. [38] suggests that tumor purity is unlikely to have an effect on gene expression skewness. However, a further exploration is challenging at this current stage because not all datasets used in the skewness study have tumor purity data available.

## Conclusions

In this article, we have demonstrated the efficacy of skewness as an indicator of biological hetrogeneity in gene pathways causally related to specific cancer cohorts. Furthermore, we have presented evidence for a link between outlying gene expression (which formes the skewed tails) and differential promoter region methylation as compared to their cohort baseline. This suggests that skewness may provide more than a gross statistic for use in comparing two datasets but possibly a metric with direct biological implications. However, we stress that our results indicate a strong correlation between those patients who exhibit gene expression in the extremities of the distribution (the tail region) and those whose corresponding gene exhibits exceptional promoter methylation only in the most extremely skewed genes. Overall, we do not claim, nor does the evidence suggest, a strict causal relationship between differential methylation and gene skewness. That said, these results suggest that skewness may provide valuable insight in the analysis of large patient cohorts beyond those analysis based on changes in gene distribution means and variances alone. We hope that future studies will investiage the specific role that methylation plays in driving the skewness apparent in gene expression distributions and their differences between cohorts. Furthermore, we suggest that future research on large patient cohorts will consider skewness and possible higher moments to glean further information from their aggregate data and to further investigate the conclusion that gene expression skewness reflects biological realities of the cohort.

## Materials and Methods

*Availability of R Code.*https://github.com/humford/epsilon. R version 3.2.3 was used for all analysis.

*Microarray datasets.* Samples were collected from primary solid tumour and recurrent solid tumour, and are log2-transformed and Lowess normalized. These data can be accessed from the TCGA Level 3 database or the legacy portal hosted by GCD. All three datasets were collected at University of North Carolina at Chapel Hill (UNC) using an Agilent 244K Custom Gene Expression Microarray (G4502A-07-3). The HapMap expression data were log2 transformed and normalized using quantile normalization [32].

*RNA-Seq datasets.* The five cancer cohorts were taken from TCGA level 3 data. All five datasets contain samples from primary solid tumour and recurrent solid tumour collected at UNC using Illumina HiSeq 2000 RNA Sequencing Version 2 Analysis, were log2-transformed and reported as fragments per kilobase of transcript per million (FPKM) mapped reads via RSEM [33]. The RNA-seq control dataset was downloaded from the 1000 Genomes project created by the Geuvadis consortium (ArrayExpress accession id E-GEUV-1). These data were collected using an Illumina HiSeq 2000, processed with GEM mapper 1.349, and log2-transformed. We transformed all RNA-seq datasets from FPKM count normalization to transcripts per kilobase of transcript per million (TPM) for our analysis.

*DNA methylation datasets.* All DNA methylation datasets were generated using an Illumina Human Methylation 450k BeadChip array. Summary intensities were extracted by the methylumi R package (v. 2.10.0 run in R v. 3.1.0). In our analysis, we transformed beta-values to M-values with a logit transformation due to advantages in statistical robustness of M-values over beta-values given the nature of our analysis [34]. The UCSC Genome Database provides annotation for Illumina 450k methylation probes including the functional region of the gene in which each methylation site is located [35]. We separated probes into three categories based on their methylation site functional region as determined by the UCSC annotations: Promoter, defined as the region 1500bp upstream of the transcriptional start site (annotated as TSS1500 including TSS200 in UCSC database); Untranslated regions (UTRs), located at both the 3’ and 5’ ends of the gene; and Body, made up of all other annotated regions (body and first exon).

*Mixture modelling.* A variable standard deviation Gaussian mixture model from the R/Bioconductor package mclust (version 5.0.2 run on R version 3.1.2) was used to cluster genes by skew difference. K = 3 mixture components were chosen to maximize the Bayesian Information Criterion (BIC) over the range 1 to 10 components and to fit the qualitative observation of three independent modes.

*Pathway over-representation analysis.* Gene enrichment was performed on the gene lists produced by the mixture model using a hypergeometric test run on each of the three gene categories produced by the mixture model corresponding to negative skew difference, negligible skew difference, and positive skew difference respectively. We used tools from the Bioconductor package GOstats (version 1.7.4, run on R version 3.1.2) [36] and the Bioconductor package KEGG.db (version 2.1 run on R version 3.1.2) [37]. P-values were adjusted for multiple testing using the Benjamini-Hochberg procedure within each mixture component, dataset and pathway module (Cellular Component, Molecular Function, Biological Process, and KEGG Pathway). Significant results were constrained at the 0.05 level after adjustment for multiple testing correction. Results are organized into four supporting files (Additional file 4, 5, 6, and 7: Table S1-S4) with comparison of cancers to control in microarray and RNA-seq respectively and comparisons between individual cancers again divided between the microarray and RNA-seq datasets.

## Supplementary information

**Additional file 1** Supplemental Figure S1. Investigating the Skewness-Mean Gene Expression Relationship for RNA-seq Datasets. Plots of the skewness measure versus the mean gene expression for the **A.** control, **B.** TCGA HNSC, **C**. TCGA LGG, **D.** TCGA LUSC.

**Additional file 2** Supplemental Figure S2. Investigating the Skewness-Mean Gene Expression Relationship for Microarray Datasets. Plots of the skewness measure versus the mean gene expression for the **A.** AML, **B.** AML (NK), **C.** control, **D.** TCGA GBM, **E.** TCGA Breast Cancer (Luminal A), **F.** TCGA OV.

**Additional file 3** Figure S3-S6. Correlation Results Between Skewness and Methylation in each Methylation Dataset. (Above) The skewness of the 500 Most significant genes plotted against the average change in methylation of their significant annotated methylation sites classified by functional region. The data are split into quadrants representing positive/negative skewness and increase/decrease in methylation. (Red points = upper left quadrant, blue = upper right, purple = lower left, green = bottom right)

**Additional file 4** Supplemental File Table S1. Microarray Cancer to Control Gene Enrichment Results. The ten most significant GOStats pathways for each subcategory comparing the microarray cancer cohorts to control. Results are separated by GOStat term function (Biological Process: BP, Cellular Component: CC, Molecular Function: MF, and KEGG), comparison group (“UP”, “DOWN”, “MIDDLE”), and by cancer cohort. https://drive.google.com/open?id=0B57SQtVF1CxkMkwzRHZpcTcwWWM

**Additional file 5** Supplemental File Table S2. Microarray Cancer to Cancer Gene Enrichment Results. The ten most significant GOStats pathways for each subcategory comparing between microarray cancer cohorts. Results are separated by GOStat term function (Biological Process: BP, Cellular Component: CC, Molecular Function: MF, and KEGG), comparison group (“UP”, “DOWN”, “MIDDLE”), and by the two cancer cohort being compared. UP refers to larger skew in the first cohort indicated. https://drive.google.com/open?id=0B57SQtVF1CxkemhMLWdBeFVZWVE

**Additional file 6** Supplemental File Table S3. RNA-Seq Cancer to Control Gene Enrichment Results. The ten most significant GOStats pathways for each subcategory comparing the RNA-Seq cancer cohorts to control. Results are separated by GOStat term function (Biological Process: BP, Cellular Component: CC, Molecular Function: MF, and KEGG), comparison group (“UP”, “DOWN”, “MIDDLE”), by cancer cohort. https://drive.google.com/open?id=0B57SQtVF1CxkUFBVQkxDcXJGWDg

**Additional file 7** Supplemental File Tables S4. RNA-Seq Cancer to Cancer Gene Enrichment Results. The ten most significant GOStats pathways for each subcategory comparing between RNA-Seq cancer cohorts. Results are separated by GOStat term function (Biological Process: BP, Cellular Component: CC, Molecular Function: MF, and KEGG), comparison group (“UP”, “DOWN”, “MIDDLE”), and by the two cancer cohort being compared. UP refers to larger skew in the first cohort indicated. https://drive.google.com/open?id=0B57SQtVF1Cxkd0VCUURhZnVoX00

**Additional file 8** Supplemental File Table S5-S8. GOStats Terms in Each Category for Classification of Differential Skew. Specific GOStats terms and pathways included in each category used to summarize gene enrichment data. https://drive.google.com/open?id=0B57SQtVF1CxkUnRDU3A4Tm1DUkUhttps://drive.google.com/open?id=0B57SQtVF1CxkZlgwdmQwSWpUOTQhttps://drive.google.com/open?id=0B57SQtVF1CxkRzlnN2s5Y1pPNjghttps://drive.google.com/open?id=0B57SQtVF1CxkVEdiYktBbkc4YWc

## Data Availability

All data used in this study is publicly available.

## Abbreviations

GBM: Glioblastoma multiforme
GO: Gene ontology
KEGG: Kyoto encyclopedia of genes and genomes
KIRC: Kidney renal clear cell carcinoma
LGG: Lower grade glioma
OV: Ovarian serous cystadenocarcinoma
SKCM: Skin cutaneous melanoma
TCGA: The cancer genome atlas
UTR: Untranslated region
