# Supplementary figures and images for: Investigating skewness to understand gene expression heterogeneity in large patient cohorts
Source: BMC Bioinformatics. 2019 Dec 20;20(Suppl 24):668. doi: 10.1186/s12859-019-3252-0 (PMC6923883; doi:10.1186/s12859-019-3252-0)

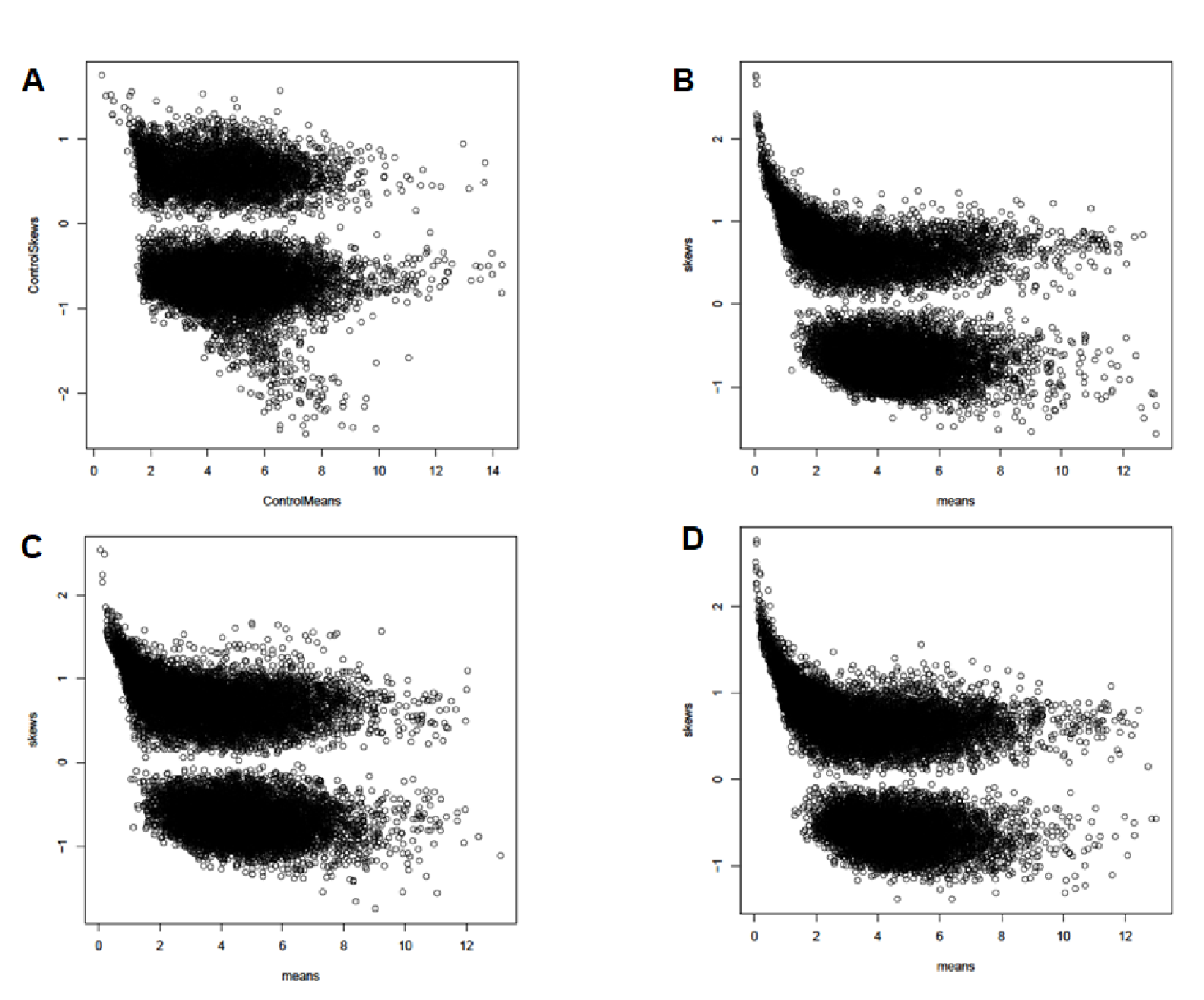

Supplement: Supplementary file 1 — Additional file 1 Supplemental Figure S1. Investigating the Skewness-Mean Gene Expression Relationship for RNA-seq Datasets. Plots of the skewness measure versus the mean gene expression for the A. control, B. TCGA HNSC, C. TCGA LGG, D. TCGA LUSC. [file 12859_2019_3252_MOESM1_ESM.png]

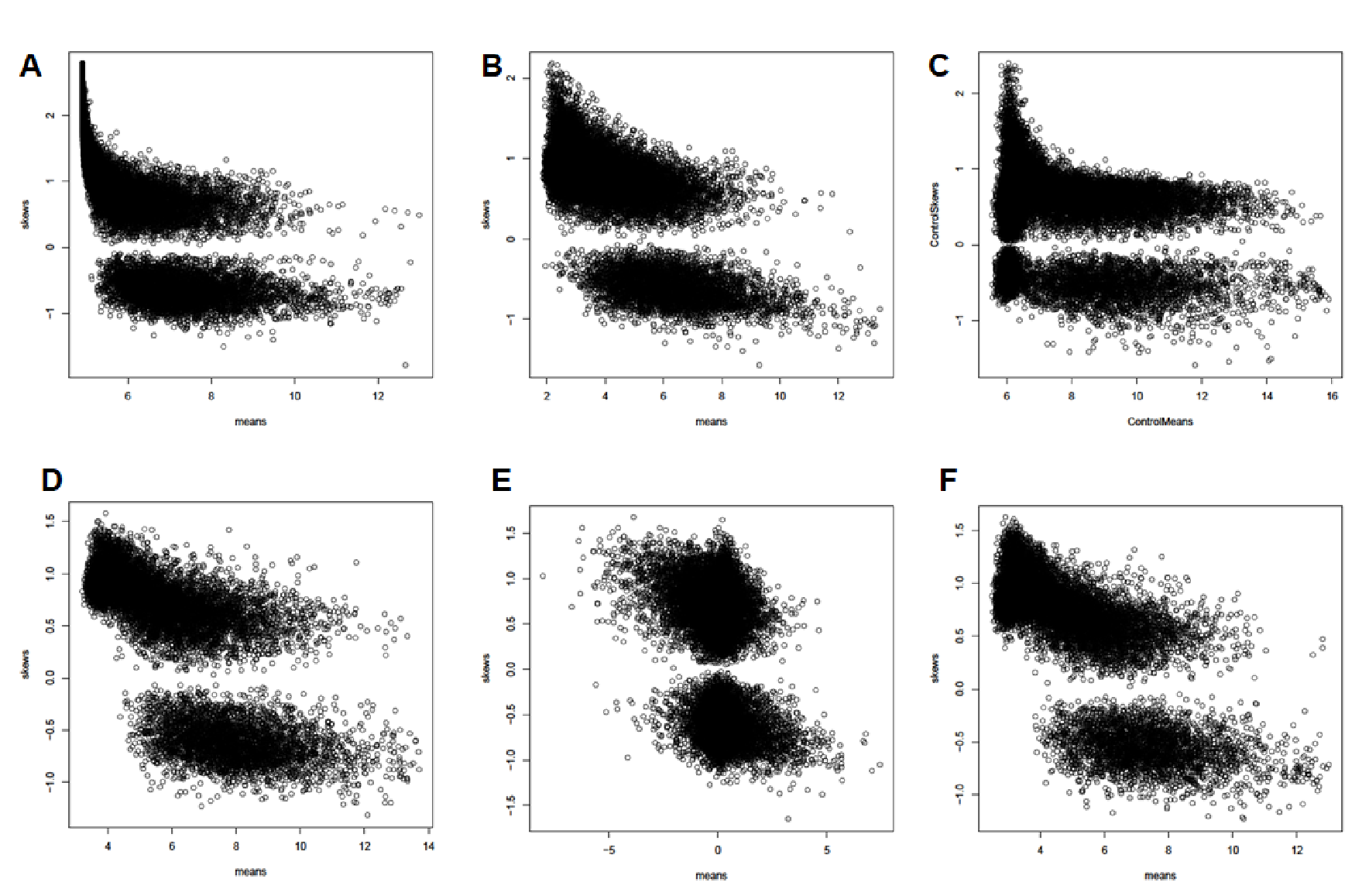

Supplement: Supplementary file 2 — Additional file 2 Supplemental Figure S2. Investigating the Skewness-Mean Gene Expression Relationship for Microarray Datasets. Plots of the skewness measure versus the mean gene expression for the A. AML, B. AML (NK), C. control, D. TCGA GBM, E. TCGA Breast Cancer (Luminal A), F. TCGA OV. [file 12859_2019_3252_MOESM2_ESM.png]
