# Supplementary material for: Investigating skewness to understand gene expression heterogeneity in large patient cohorts
Source: BMC Bioinformatics. 2019 Dec 20;20(Suppl 24):668. doi: 10.1186/s12859-019-3252-0 (PMC6923883; doi:10.1186/s12859-019-3252-0)
Supplement: Supplementary file 3 — Additional file 3 Figure S3-S6. Correlation Results Between Skewness and Methylation in each Methylation Dataset. (Above) The skewness of the 500 Most significant genes plotted against the average change in methylation of their significant annotated methylation sites classified by functional region. The data are split into quadrants representing positive/negative skewness and increase/decrease in methylation. (Red points = upper left quadrant, blue = upper right, purple = lower left, green = bottom right) [file 12859_2019_3252_MOESM3_ESM.pdf]

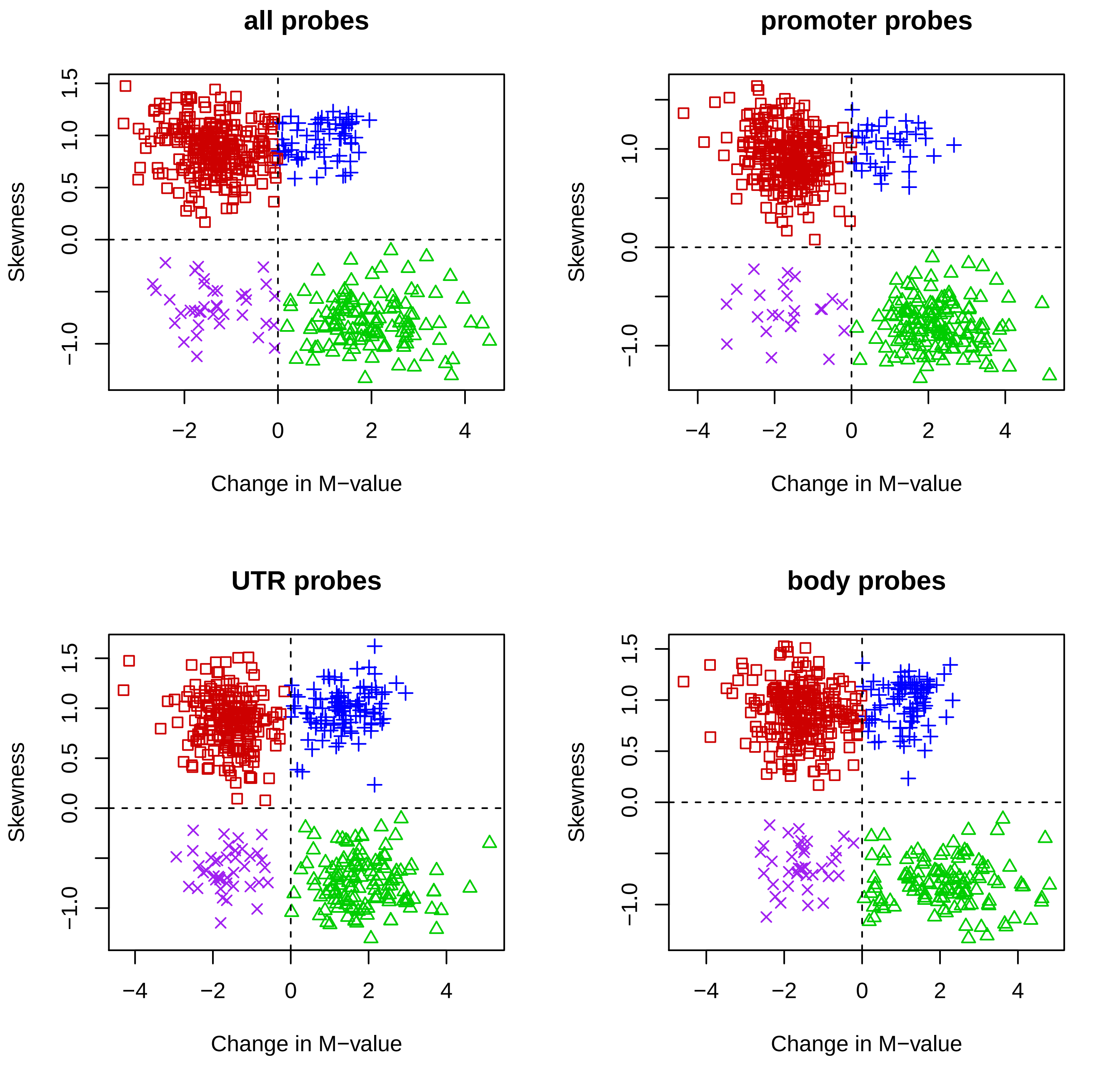

all probes
promoter probes
UTR probes
body probes
Skewness
Change in M−value
1.5
1.0
0.5
0.0
−1.0
−4
−2
0
2
4

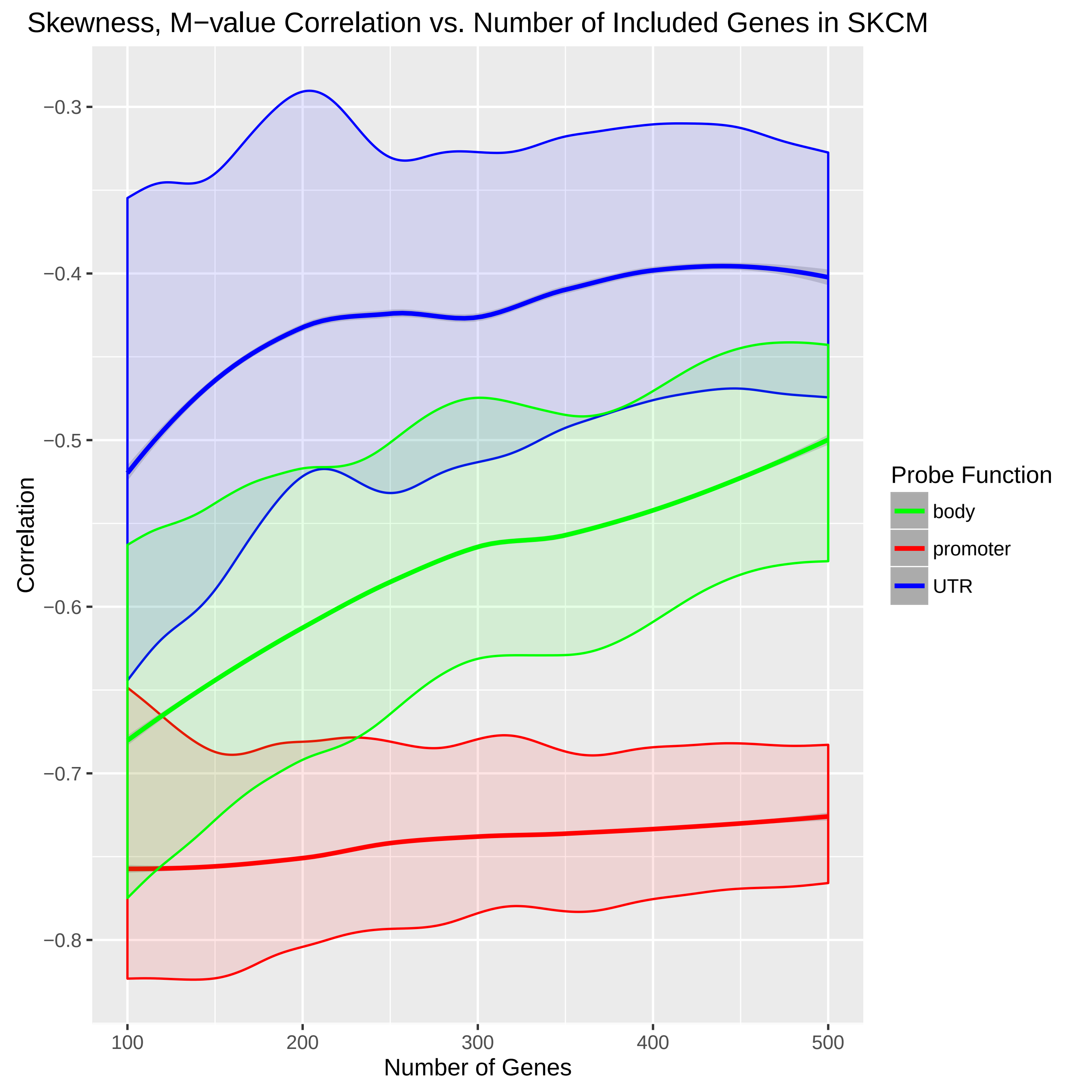
Skewness, M−value Correlation vs. Number of Included Genes in SKCM
Correlation
Number of Genes
−0.3
−0.4
−0.5
−0.6
−0.7
−0.8
100
200
300
400
500
Probe Function
body
promoter
UTR
